# Intermittent theta burst stimulation for negative symptoms of schizophrenia—A double-blind, sham-controlled pilot study

**DOI:** 10.1038/s41537-021-00138-3

**Published:** 2021-02-12

**Authors:** Rémy Bation, Charline Magnin, Emmanuel Poulet, Marine Mondino, Jérôme Brunelin

**Affiliations:** 1 INSERM, U1028; CNRS, UMR5292; Lyon Neuroscience Research Center, PSYR2 Team, Lyon, France; 2 University Lyon 1, Villeurbanne, France; 3 Centre Hospitalier Le Vinatier, Bron, France; 4 Psychiatry Unit, Wertheimer Hospital, CHU Lyon, Bron, France; 5 Psychiatry Emergency Unit, Edouard Herriot Hospital, CHU Lyon, Lyon, France

**Keywords:** Schizophrenia, Neural circuits

## Abstract

Optimal noninvasive brain stimulation parameters for the treatment of negative symptoms of schizophrenia remain unclear. Here, we aimed to investigate the clinical and biological effects of intermittent theta burst transcranial magnetic stimulation (iTBS) in patients with treatment-resistant negative symptoms of schizophrenia (NCT00875498). In a randomized sham-controlled 2-arm study, 22 patients with schizophrenia and treatment-resistant negative symptoms received 20 sessions of either active (*n* = 12) or sham (*n* = 10) iTBS. Sessions were delivered twice a day on 10 consecutive working days. Negative symptom severity was assessed 5 times using the Scale for the Assessment of Negative Symptoms (SANS): before iTBS, after iTBS, and 1, 3, and 6 months after iTBS. As a secondary objective, we explored the acute effects of iTBS on functional connectivity of the left dorsolateral prefrontal cortex (DLPFC) using seed-based resting-state functional connectivity MRI (rsFC fMRI) images acquired before and after iTBS. Active iTBS over the left DLPFC significantly decreased negative symptoms severity compared to sham iTBS (*F*_(3,60)_ = 3.321, *p* = 0.026). Post hoc analyses revealed that the difference between groups was significant 6 months after the end of stimulation sessions. Neuroimaging revealed an increase in rsFC between the left DLPFC and a brain region encompassing the right lateral occipital cortex and right angular gyrus and a right midbrain region that may encompass dopamine neuron cell bodies. Thus, iTBS over the left DLPFC can alleviate negative symptoms of schizophrenia. The effect might be driven by significant modulation of dopamine transmission.

## Introduction

Schizophrenia is a severe and frequent mental disorder usually characterized by negative, positive and disorganization symptoms. Negative symptoms of schizophrenia (NSS) classically include affective flattening or blunting, alogia, asociality, anhedonia and avolition. NSS are highly disabling, and their treatment is a major challenge to achieve recovery in patients with schizophrenia^1^. NSS are particularly resistant to pharmacological medication^2,3^, and approximately 30% of patients still experience NSS even during treatment with antipsychotic drugs at adequate doses and durations^4^. To develop new therapeutic approaches, studies have focused on exploring the neurobiological underpinnings of NSS.

At a structural level, imaging studies have reported that the volume of prefrontal regions, including orbitofrontal, medial and lateral prefrontal cortices, was inversely correlated with NSS severity^5,6^. Interestingly, a white matter volume decrease was observed in prefrontal areas in patients with NSS and was associated with higher levels of NSS^7^. At a functional level, studies using positron emission tomography (PET) and single photon emission computed tomography (SPECT) found that NSS were strongly associated with decreased frontal and prefrontal metabolism at rest or during activation^8,9^. Specific prefrontal areas, in particular, the dorsolateral prefrontal cortex (DLPFC), have been identified^10^. More recent studies have suggested that patients with NSS have structural and functional impairments in prefrontal areas and that the connectivity between those areas and the rest of the brain, including striatal regions, is also impacted^11^.

Repetitive transcranial magnetic stimulation (rTMS) is a neuromodulation technique that can modulate the activity and connectivity of the brain, including when applied over the DLPFC^12^. rTMS uses an external magnetic field applied directly on the scalp to modulate the electrical activity of neurons located under the stimulation coil. Repeated stimulations induce changes in cortical excitability that outlast the stimulation period. In patients with NSS, applying rTMS with high-frequency (HF) stimulation over the left DLPFC has shown promise^13^. However, although some positive studies have been published that led European guidelines to conclude that there is possible efficacy of HF rTMS over the left DLPFC for NSS^14^, large negative multicenter randomized studies are also available (e.g., ref. ^15^). To explain these discrepancies between studies, one can hypothesize that rTMS parameters have not been optimized. Intermittent theta burst stimulation (iTBS) is a rTMS protocol that consists of delivering bursts of 3 TMS pulses at high frequency (50 Hz) every 200 ms for 2 s every 10 s (i.e., intermittently). This form of stimulation allows delivering a more important number of rTMS pulses in a shorter period of time than standard HF rTMS protocols and is known to produce long-lasting effects on cortical excitability^16^. In a recent randomized controlled trial comparing 10 Hz rTMS, 20 Hz rTMS and iTBS in patients with NSS, Zhao and colleagues^17^ showed that iTBS resulted in a significantly larger reduction in NSS than classic HF rTMS protocols. Taken together, these studies claimed that iTBS over the left DLPFC should be used to decrease NSS, but the results have to be replicated. Moreover, a better understanding of the biological effect of iTBS in patients with NSS would help us to optimize stimulation efficacy.

Here, we hypothesized that repeated sessions of iTBS over the left DLPFC would result in alleviation of NSS by modifying functional connectivity of the DLPFC. The aim of this work was (1) to investigate the clinical effects of iTBS targeting the left DLPFC on NSS and (2) to explore functional connectivity modifications induced by iTBS with resting-state functional connectivity (rsFC) MRI.

## Results

As shown in Table 1, the active and sham groups did not significantly differ in demographics, electrophysiological measures and baseline clinical ratings.

**Table 1:** Demographic and clinical baseline characteristics of the patients included in the study.

	Active iTBS		Sham iTBS		
	Mean	SD	Mean	SD	*p*
*n*	12		10		
Sex (male) (%)	100		90		0.45
Left-handers (% right)	92		80		0.57
Age (years)	42.33	9.44	41.60	12.63	0.88
Educational level (years)	11.45	2.54	12.10	2.81	0.59
Duration of illness (years)	15.00	5.86	17.11	15.37	0.67
Medication (eq cpz mg/day)	325.00	206.23	389.10	170.74	0.44
PANSS total score	78.83	7.96	71.50	14.58	0.15
PANSS positive subscale	10.50	3.06	9.70	1.83	0.48
SANS	77.75	13.00	71.10	21.18	0.38
Depression (CDSS)	4.00	2.63	1.89	2.67	0.09
Simpson-Angus Scale	1.25	1.54	4.10	4.84	0.07
Resting motor threshold (%)	54.33	8.41	50.70	7.62	0.31

*CDSS* Calgary depression scale for schizophrenia, *eq cpz* chlorpromazine equivalent, *PANSS* positive and negative syndrome scale, resting motor threshold is expressed as a % of maximal stimulator output; *SANS* scale for the assessment of negative symptoms. Student’s t tests were used to compare demographic and clinical characteristics between the two groups. Sex and handedness proportion differences were assessed using chi-square tests. No differences were observed between patients in the active and sham groups.

### Effects of iTBS on negative symptoms

**Fig. 1:**
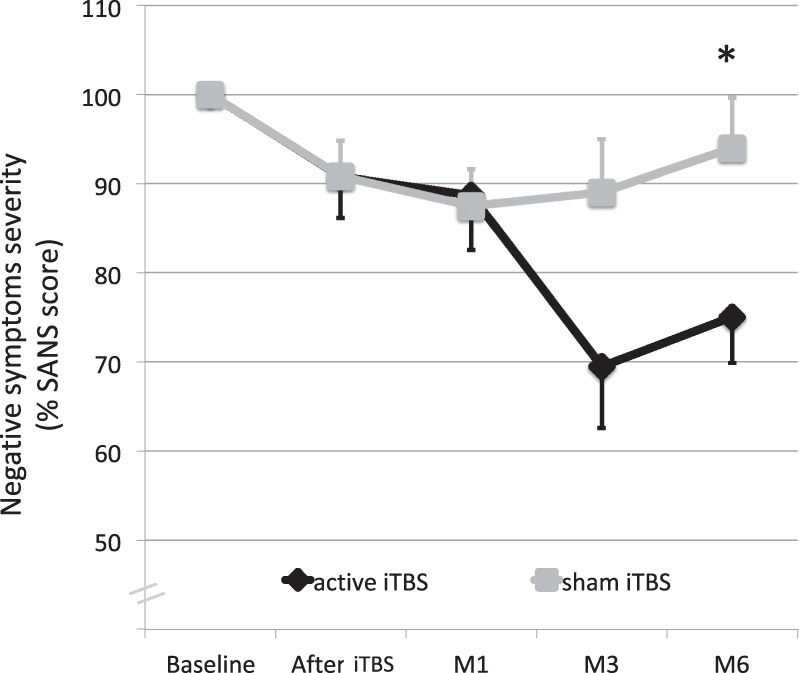
Clinical effects of iTBS on negative symptoms. Changes in negative symptoms (SANS scores) over time in the active and sham groups receiving intermittent theta burst stimulation (iTBS). M1, M3, M6 represent follow-up assessments at 1, 3, and 6 months after the end of iTBS treatment, respectively. Error bars represent standard errors of the mean.

Repeated measures ANOVA on SANS scores revealed a significant interaction between time and group (*F*_(3,60)_ = 3.32, *p* = 0.026). After the end of the trial (6-month follow-up), a mean decrease of 26.72% (standard error of the mean (sem) = 5.50%) in the SANS score was observed in the active group, and a mean decrease of 6.07% (5.91%) was observed in the sham group. The post hoc comparisons showed a significant difference in SANS scores between the active group and the sham group only at the endpoint time sixth months after the iTBS sessions (*p* = 0.019). The effect size was large according to the standardized effect sizes (Cohen’s *d* = 1.09). The changes in total SANS scores throughout the study period is presented in Fig. 1.

To rule out a potential effect of the severity of depression at baseline on our results, we added CDSS scores as a covariate in our analysis. We found no significant effect of CDSS (*F*_(1,18)_ = 0.83, *p* = 0.37) or time X CDSS interaction (*F*_(3,54)_ = 1.34, *p* = 0.27). In contrast, the effect of the group X time interaction remained significant (*F*_(3,54)_ = 5.37, *p* = 0.003).

### Effects of iTBS on other clinical dimensions

Repeated measures ANOVA on PANSS total scores revealed no significant interaction between time and group (*F*_(3,60)_ = 1.62, *p* = 0.20). No interaction was observed regarding PANSS subscale scores: PANSS positive score (*F*_(3,60)_ = 0.61, *p* = 0.61), PANSS negative score (*F*_(3,60)_ = 1.62, *p* = 0.19), and PANSS general psychopathology score (*F*_(3,60)_ = 1.05, *p* = 0.38).

### Tolerability and blinding

The intervention was well tolerated, and no seizures or other life-threatening events occurred. In both groups, mild headaches were reported during the stimulation sessions. There were two serious adverse events during the follow-up period: two patients from the sham group displayed an exacerbation of positive symptoms requiring hospitalization.

Treatment conditions were not correctly classified by either the patients (chi^2^_(1, *N* = 22)_ = 0.22, *p* = 0.64) or the clinical raters (chi^2^_(1, *N* = 22)_ = 1.77, *p* = 0.18).

### Effects of iTBS on left DLPFC seed-based rsFC

**Fig. 2:**
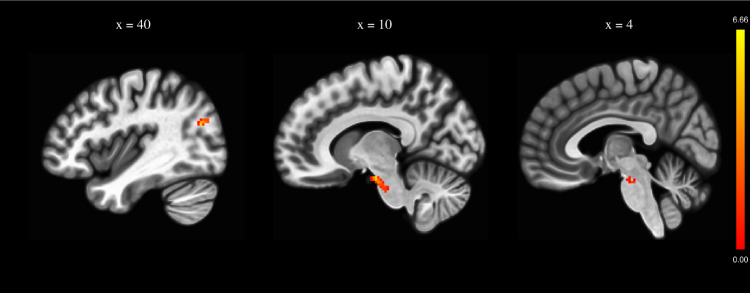
Biological effects of iTBS on brain connectivity. Brain regions with significant changes in resting-state functional connectivity (rsFC) with the left dorsolateral prefrontal cortex after 20 sessions of active intermittent theta burst stimulation (iTBS, *N*=10) compared to sham iTBS (*N*=7). The rsFC changes (post-iTBS minus pre-iTBS) were compared between the active and sham groups using two-sample T tests. The results were thresholded at an uncorrected voxel-level threshold of *p*<.001 with an FWE-corrected *p*<0.05 (minimum *K*=91).

Among the 22 patients, imaging data from 17 patients were available and analyzed (10 in the active group and 7 in the sham group). Between-group analysis showed no significant rsFC difference between the two groups at baseline. Comparisons of rsFC changes (post-iTBS minus pre-iTBS) between the two groups revealed that rsFC significantly increased between the left DLPFC seed and the right brain stem/midbrain (MNI coordinates *x*, *y*, *z* = 10, −12, −20; *k* = 98 voxels; *T* = 6.66; *Z* = 4.48; *p* < 0.001) and between the left DLPFC seed and a cluster encompassing the right superior lateral occipital cortex and the right angular gyrus (*x*, *y*, *z* = 40, −70, 24; *k* = 91 voxels; *T* = 6.66; *Z* = 4.47; *p* < 0.001) after active iTBS compared to sham iTBS (see Table 2 and Fig. 2).

**Table 2:** Brain regions showing significant changes in dorsolateral prefrontal resting-state functional connectivity after active vs. sham stimulation (active stimulation (post-pre) > sham stimulation (post-pre) contrast).

Brain region	BA	Cluster size	Peak coordinates			*T*	*z*
			*x*	*y*	*z*		
Right brainstem/midbrain	–	98	10	−12	−20	6.66	4.48
Right superior lateral occipital cortex/angular gyrus	19/39	91	40	−70	24	6.66	4.47

*BA* Brodmann area. Coordinates are given in MNI. The results are thresholded at an uncorrected voxel-level threshold of *p* < .001 combined with a FWE-corrected *p* < 0.05 at the cluster level.

## Discussion

In this study, we investigated the clinical and biological effects of iTBS targeting the left DLPFC on NSS. We reported a significant effect of iTBS over sham, and the improvement in NSS was statistically significant at 6 months after the treatment, with a 26% decrease in the SANS score in the active group. These results are consistent with recent meta-analyses that emphasized the efficacy of HF rTMS on NSS in patients with schizophrenia^13,18^.

Clinical improvement was observed 3 months after the end of the stimulation sessions but became statistically significant only at the evaluation time point 6 months after the end of the iTBS regimen. This long-term delayed response was unexpected but corroborated previous rTMS studies that also reported a delayed effect of rTMS on NSS. For instance, Li and colleagues^19^ observed a significant difference in SANS scores after rTMS treatment between active and sham groups 8 weeks after the end of stimulation sessions but not after 4 weeks. In patients with major depression, a delayed clinical effect was also observed two weeks after the end of an iTBS protocol, suggesting that iTBS itself could have a delayed effect mechanism^20^. In addition to the potential delayed effects of rTMS, negative symptoms are also slow to change, as engaging in more relationships and social activities can take time to establish, which is why the recommended duration of trials on negative symptoms is 6 months^21^. Another possible explanation is that our population was quite old for a population of patients with schizophrenia (more than 40 years old on average in both groups). This pathology typically starts in early adulthood, but we included resistant patients who had already had other lines of treatment and were therefore older. Older patients might have longer symptom durations and less plasticity, which could explain the delayed response.

The analysis of PANSS negative scores did not reveal any significant modification following stimulation. This is in line with Wobrock and colleagues^15^ who published negative results in a multicenter sham-controlled study including 175 patients who received either active or sham rTMS over the left DLPFC and evaluated the negative symptoms with the PANSS. This is also in line with results from Dlabac-de Lange and colleagues^22^ reporting significant effects of rTMS on NSS measured with the SANS but no significant changes in NSS assessed with the PANSS negative symptom scores. In Shi and colleagues’ meta-analysis^18^, the effect size of rTMS on negative symptoms in sham-controlled trials was 0.80 when NSS were measured by the SANS and 0.41 when measured by the PANSS, suggesting that the SANS is more sensitive to clinical changes and provides better effect size in longitudinal studies. The SANS, with its 25 items divided into 5 subscales investigating specific dimensions of NSS (affective flattening or blunting, alogia, avolition-apathy, anhedonia-asociality, and attention), seems to be more specific and sensitive than the PANSS in measuring NSS in research studies. New scales assessing NSS, such as the Clinical Assessment Interview for Negative Symptoms (CAINS^23^) and the self-evaluation of negative symptoms^24^, have been recently developed using a data-driven iterative process to improve reliability and validity^25^. It would have been interesting to evaluate the effect of iTBS using these scales, as well as directly evaluate quality of life based on functional impairments.

Regarding the imaging results from this study, the rsFC changes observed in the active group compared to the sham group included an increased connectivity between the left DLPFC and the midbrain region that may encompass dopamine (DA) neurons of the ventral tegmental area (VTA) and an increased connectivity between the left DLPFC and the right superior lateral occipital gyrus/angular gyrus (inferior parietal gyrus). Among patients with schizophrenia, the functional connectivity between the DLPFC and midbrain region, including the VTA, is known to be significantly different compared to healthy subjects^26^. The interrelation between the DLPFC and VTA may allow goal representation and maintenance and mediate the anticipated reward (wanting) function^27,28^. Anticipated reward has been linked to NSS^29^. A restored connectivity between the DLPFC and VTA could be the neurobiological correlate of an improved ability to represent anticipated reward and improve motivational deficits in schizophrenia. A recent randomized, sham-controlled, crossover study found that DLPFC iTBS treatment modulated the reward system, based on anhedonia severity, in patients with major depression disorder^30^. In two case studies in patients with NSS receiving iTBS over the left DLPFC, we previously reported that iTBS may induce DA release in the striatum^31^ and modulate glutamine/glutamate (Glx) concentrations in the DLPFC^32^. Taken together, these studies suggest that iTBS could modulate PFC-VTA connectivity and striatal DA release in patients with NSS, leading to clinical improvements. Furthermore, we reported that active iTBS increased rsFC between the left DLPFC and a cluster at the junction of the right angular gyrus and the superior division of the lateral occipital cortex. The angular gyrus has a critical contribution to changes in global brain connectivity in response to changing environmental demands. More specifically, the angular gyrus has emerged as a cross-modal hub where converging multisensory information is combined and integrated to allow comprehensive and give sense to events, manipulate mental representations, and reorient attention to relevant information^33^. Interestingly, dysfunctions in the angular gyrus have been proposed in a neurobiological model of social deficits and ego disturbances observed in schizophrenia^34^. In addition, the DLPFC, the angular gyrus and the superior lateral occipital cortex are part of the frontoparietal network, a network associated with higher cognitive functions, which showed decreased functional connectivity in patients with schizophrenia^35^. Increasing connectivity between these regions may contribute to improving cognitive functions and decreasing symptoms of schizophrenia. Here, we observed that left DLPFC stimulation induced rsFC changes between the DLPFC and regions of the right hemisphere. Only a few studies have specifically investigated the impact of DLPFC-rTMS in patients with NSS. In healthy volunteers, interhemispheric effects have also been suggested with HF rTMS over the right DLPFC inducing a significant decrease in seeded functional connectivity of the targeted region with the contralateral hippocampus while participants performed in a working memory task^36^. Further neuroimaging studies are needed to better understand the relationship between the iTBS-induced changes and the clinical improvements in NSS. For instance, future studies should investigate the correlations between observed clinical changes and changes in DLPFC connectivity strength at an individual level.

The power of the results is limited by the small sample size. However, despite the small number of participants in the study, the careful selection of participants led to a relative homogeneity of symptoms and the large effect sizes support our conclusions and recent clinical studies^37^. This rather small sample did not allow us to investigate potential differences between responders and nonresponders to the iTBS intervention, although this is a major challenge in the TMS field. Another limitation of our study is that the patients were taking different antipsychotic medications. It would have been better to include patients with the same treatment to improve the comparability and to better explore potentiation effects. For instance, Wagner and colleagues^38^ found that rTMS and clozapine could have a good potentiation effect on NSS. Moreover, the sex proportion was skewed towards males, thereby, contributing to the limitation of generalizability of current results. As in several other studies in patients with schizophrenia, we included a mixed sample with both right-handers and left-handers. While little is known regarding the effect of handedness on the TMS-induced effect on NSS, a recent meta-analysis in patients with major depression concluded that handedness did not influence rTMS effects on clinical symptoms^39^. This point merits further investigation in schizophrenia since abnormalities of laterality have been repeatedly reported in patients.

Despite these limitations, this study is one of the few randomized controlled studies to evaluate the clinical and biological effects of iTBS in persistent NSS. The population was carefully selected to avoid any confounding factors (depressive symptoms, positive symptoms, recent change in pharmaceutical treatment, etc.) in accordance with guidelines on clinical trials designed for NSS. At baseline, extrapyramidal side effects measured with the SAS were mild in both groups, which confirmed the primary nature of NSS in the included patients. Our results remained significant when baseline CDSS scores were added as a covariate in the variance analysis, indicating that the slight difference in depression severity at baseline did not influence iTBS effects on NSS while iTBS over the DLPFC is known to decrease depressive symptoms including anhedonia in patients with major depression^20^. The blinding integrity was confirmed for both patient and clinician raters. Our study indicated that iTBS over the DLPFC is safe in a population of patients with persistent NSS. The effectiveness of iTBS on NSS can be considered large (*d* = 1.09), although the included population displayed characteristics that have been associated with lower response rates to rTMS (e.g., older age and longer duration of disease). These effects translate into real clinical benefits for patients from the active group with a mean decrease is SANS scores of more than 25% that is consensually accepted as a meaningful decrease^40^. However, future studies should investigate quality of life and functioning of patients with NSS receiving iTBS with specific clinical scales and ecological evaluation. This efficiency could be related to the high number of pulses delivered (19,800) delivered at a known efficient intensity set at 80% RMT^41^ based on an iTBS protocol that allows fast and safe delivery of TMS pulses. Indeed, among others, one advantage of iTBS compared to standard rTMS protocols is the decrease in financial and time burdens of rTMS. Positive symptoms were not worsened by the intervention, in contrast to the modest worsening of positive symptoms observed in a recent meta-analysis on rTMS trials for NSS^42^.

In sum, iTBS targeting the left DLPFC shows promising results in the long-term treatment of persistent negative symptoms. iTBS of the left DLPFC may restore the connectivity of the DLPFC with areas involved in the representation of pleasure and the self and with areas involving mesocorticolimbic DA transmission.

## Methods

In a randomized double-blind sham-controlled study, twenty-two patients with schizophrenia were randomized to receive either active (*n* = 12) or sham (*n* = 10) rTMS treatment. The clinical raters and the participants were blinded to the treatment conditions. A randomization list was generated with the ALEA function in an Excel file by the sponsor of the study. The randomization was performed by block (block size of four: 2 active, 2 sham). There was no stratification. After inclusion of each patient by the investigator, the TMS operator called the sponsor to obtain the random assignment of the patient (active or sham). The TMS operator was not blinded to the treatment condition, that is, only the patient and the rater were blinded.

### Participants

**Fig. 3:**
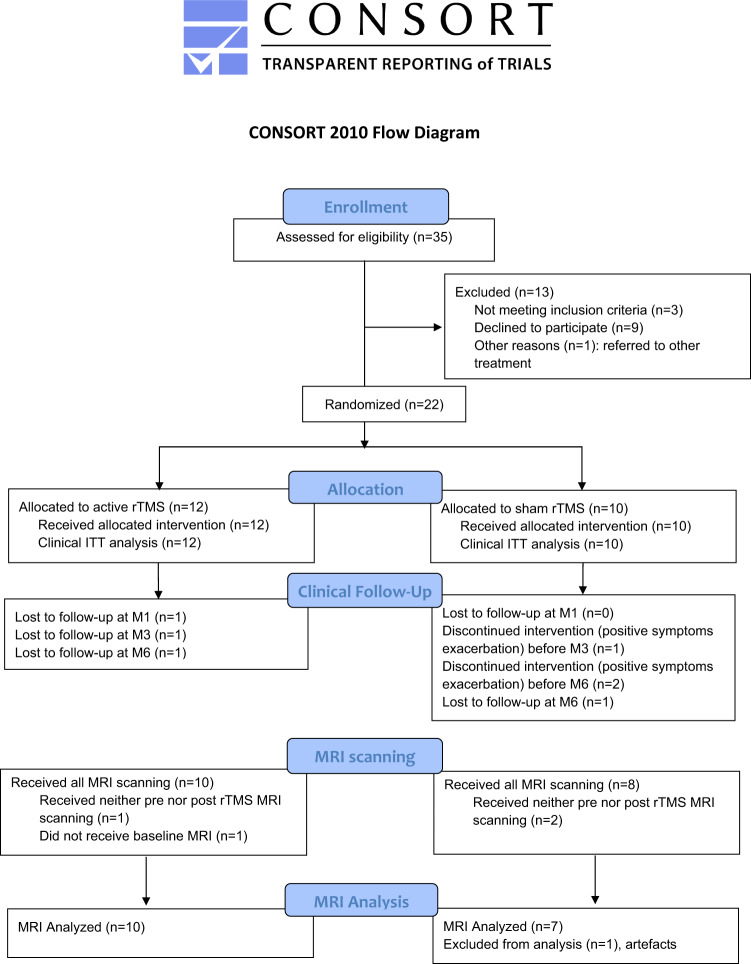
Patients selection. CONSORT flow chart diagram of the study.

A total of 35 patients were screened for eligibility. Nine patients declined to participate, three did not meet the inclusion criteria, and one patient was referred to another treatment (please see the CONSORT flow chart diagram shown in Fig. 3). The final analyzed sample consisted of 22 outpatients between 22 and 65 years old meeting the DSM-IV-TR criteria for schizophrenia. NSS were resistant to at least two antipsychotic medications at effective dosages for at least 6 weeks^43^. According to the inclusion criteria, patients experienced prominent NSS (Positive and Negative Syndrome Scale (PANSS^44^) negative subscale ≥20, with at least 2 items ≥4) without showing a > 20% reduction in PANSS negative subscores between the preinclusion visit (at least one month before inclusion) and the inclusion visit. They also presented neither clinically significant positive symptoms (no positive item ≥4 and PANSS positive subscale <20) nor depressive symptoms as assessed by a Calgary Depression Scale for Schizophrenia (CDSS^45^) score <9. The exclusion criteria were as follows: clinically relevant psychiatric comorbidity (including current misuse of or dependence on illegal drugs or alcohol), concomitant treatment with anticonvulsant drugs or benzodiazepines (lorazepam >2 mg/day or equivalent), history of epileptic seizures, previous treatment with rTMS, a contraindication for rTMS, clinically relevant unstable medical conditions, involuntary hospitalization, or pregnancy. Antipsychotic medication had to be stable for at least 4 weeks before study inclusion and remained stable throughout the study. Extrapyramidal symptoms were assessed in each group with the Simpson-Angus Scale (SAS)^46^, a 10-item scale with item scores ranging from 1 to 5, designed to assess the presence and severity of rigidity and bradykinesia.

The protocol was reviewed and approved by a local ethics committee (comité de protection des personnes- CPP Sud Est 6–AFSSAPS n° 2008-A00558-47). All subjects gave their written informed consent after a detailed description of the study by the investigators from our Brain Stimulation Unit (CH Le Vinatier, psychiatric hospital, France). Details of the study were registered on ClinicalTrials.gov (identifier NCT00875498) on April 3, 2009.

### rTMS procedure

Patients received 20 sessions (2 sessions per working day for 2 weeks) of either active or sham stimulation delivered by a Mag Pro X100 (Magventure, Mag2Health, France). The iTBS protocol consisted of bursts containing 3 pulses at 50 Hz repeated at 200-ms intervals for 2 s (i.e., at 5 Hz). A 2-s train of iTBS was repeated every 10 s for a total of 990 pulses per session (adapted from^47^). The intensity of stimulation was set at 80% of the resting motor threshold (RMT) intensity. During the first session, the RMT intensity was measured and corresponded to the minimal intensity that resulted in a visual motor response of the thumb when stimulating the contralateral motor cortex. Stimulations were delivered over the left DLPFC (middle frontal gyrus, junction between Brodmann areas 9 and 46) by placing a figure-8 coil 6 cm anterior to the scalp position corresponding to the motor cortex (the hot spot where the motor threshold was defined)^48^. The patients in the sham group received the same iTBS regimen, but stimulations were delivered using a commercial figure-8 sham coil.

### Clinical assessment and analysis

The severity of NSS was assessed 5 times throughout the study period with the SANS^49^: at baseline, after the iTBS sessions and then after 1, 3, and 6 months after the end of iTBS. The effect of iTBS on overall schizophrenia symptoms was assessed using the PANSS.

Student’s t tests were used to compare demographic and clinical characteristics between the two groups at baseline. Sex and handedness proportion differences were assessed using chi-square tests.

All analyses were undertaken on a strict intention-to-treat sample of the evaluable patients defined in the protocol as patients with a baseline assessment and at least one post-rTMS score. Changes (% change from baseline) in the SANS total score over the 6-month follow-up period were compared using repeated measures analysis of variance (ANOVA), including terms for group, time, and time by group interaction. The effects on other clinical measures (PANSS) were analyzed in the same manner. The analyses were conducted in a last observation carried forward (LOCF) manner through the final endpoint. For significant interactions, Fisher’s least significant difference post hoc tests were used to determine the timing of treatment differences. Standardized effect sizes (Cohen’s d) were calculated for significant results by dividing the estimated group difference by a measure of background variability (standard deviation of baseline score) as suggested by Cohen^50^. An effect size of 0.2 was considered small; 0.5, medium; and 0.8, large. All tests were conducted with two-sided significance levels (α = 0.05).

Blinding integrity was controlled by using a chi-square test on the patients’ and blinded raters’ ability to correctly guess the treatment allocation.

### Functional MRI (fMRI) data acquisition and preprocessing

Images were acquired at the CERMEP imaging center of Lyon (France) on a 1.5 T Magnetom Siemens scanner with a standard 8-channel head coil. A 3-dimensional (3D) T1-weighted anatomical scan covering the whole brain volume was initially acquired with the following parameters: 176 transverse slices, repetition time (TR) = 1970 ms, echo time (TE) = 3.93 ms, field of view = 256 mm², and voxel size = 1 mm^3^.

The participants underwent 2 resting-state fMRI scans: the first was acquired on the Friday before starting the iTBS sessions, and the second was acquired 1 h after the end of the 20th iTBS session. The fMRI resting-state data were acquired using a gradient-echo T2*-weighted echo-planar imaging (EPI) sequence with the following parameters: 5 min, 120 volumes, 29 transverse slices, TR = 2500 ms, TE = 50 ms, field of view = 220 mm², and voxel size = 3.438 × 3.438 × 4 mm. During the resting sequence, the participants remained in a state of wakeful rest; they were instructed to stay awake and to lie still with their eyes open, fixating on a white cross presented at the center of the visual field. They were instructed to not fall asleep and to think of nothing in particular. All participants wore headphones to attenuate scanner noise.

The fMRI resting-state data were preprocessed and analyzed using the CONN toolbox (v18.b, https://www.nitrc.org/projects/conn) in conjunction with SPM12. After the removal of the first 5 volumes, functional images were realigned (motion estimation and correction), unwarped, and slice-time corrected. Outlier detection was conducted using the artifact detection toolbox. The volumes in which the normalized z-scores from global mean signal intensity exceeded 5 or the composite motion was over 0.9 mm were identified as outliers. Then, functional images were segmented and spatially normalized into the standard MNI space (Montreal Neurological Institute, Canada) and smoothed using an 8-mm full-width half-maximum (FWHM) Gaussian kernel. A segmentation and normalization of anatomical scans was conducted to obtain white matter and cerebrospinal fluid masks for each participant. Denoising included the linear regression of several confounding parameters, including the six motion parameters derived from spatial-motion correction and their first-order derivatives, as well as the BOLD signal from the CSF and white matter masks (5 components each) using the anatomical CompCor approach. Motion outliers detected by the artifact detection toolbox were censored. Functional images were bandpass filtered using a temporal filter of 0.008 to 0.09 Hz and a linear detrending was performed.

### Seed-based rsFC analysis

For each subject, correlations between the extracted time course of an a priori seed region with other brain voxels were computed. A 10-mm spherical seed region corresponding to the left DLPFC (MNI coordinates: *x* = −43; *y* = 33; *z* = 36) was defined according to Mylius and colleagues^51^. At the first-level, Fisher’s z-transformed Pearson’s correlation coefficients were computed between the mean time-series averaged across seed voxels that were within the estimated gray matter mask for each subject and the time course of all of the other voxels. At the second-level, between-group analyses were performed to compare the left DLPFC functional connectivity changes (post-iTBS minus pre-iTBS) between active and sham groups using two-sample T tests. The results were thresholded at an uncorrected voxel-level threshold of *p* < 0.001 with a familywise error (FWE)-corrected *p* < 0.05 at the cluster level to correct for multiple comparisons.

### Reporting summary

Further information on research design is available in the Nature Research Reporting Summary linked to this article.

## Supplementary information

REPORTING SUMMARY

## Data Availability

The data that support the findings of this study are available from the corresponding author (J.B.) upon reasonable request.
