# Supplementary material for: Intermittent theta burst stimulation for negative symptoms of schizophrenia—A double-blind, sham-controlled pilot study
Source: NPJ Schizophr. 2021 Feb 12;7:10. doi: 10.1038/s41537-021-00138-3 (PMC7880987; doi:10.1038/s41537-021-00138-3)
Supplement: Supplementary file 1 — REPORTING SUMMARY [file 41537_2021_138_MOESM1_ESM.pdf]

nature research

Corresponding author(s): BRUNELIN J

Last updated by author(s): Dec 10, 2020

Reporting Summary

Nature Research wishes to improve the reproducibility of the work that we publish. This form provides structure for consistency and transparency in reporting. For further information on Nature Research policies, see our Editorial Policies and the Editorial Policy Checklist.

Statistics

For all statistical analyses, confirm that the following items are present in the figure legend, table legend, main text, or Methods section.

n/a	Confirmed	
☐	☒	The exact sample size (n) for each experimental group/condition, given as a discrete number and unit of measurement
☐	☒	A statement on whether measurements were taken from distinct samples or whether the same sample was measured repeatedly
☐	☒	The statistical test(s) used AND whether they are one- or two-sided<br>*Only common tests should be described solely by name; describe more complex techniques in the Methods section.*
☐	☒	A description of all covariates tested
☐	☒	A description of any assumptions or corrections, such as tests of normality and adjustment for multiple comparisons
☐	☒	A full description of the statistical parameters including central tendency (e.g. means) or other basic estimates (e.g. regression coefficient) AND variation (e.g. standard deviation) or associated estimates of uncertainty (e.g. confidence intervals)
☐	☒	For null hypothesis testing, the test statistic (e.g. F, t, r) with confidence intervals, effect sizes, degrees of freedom and P value noted<br>*Give P values as exact values whenever suitable.*
☒	☐	For Bayesian analysis, information on the choice of priors and Markov chain Monte Carlo settings
☒	☐	For hierarchical and complex designs, identification of the appropriate level for tests and full reporting of outcomes
☐	☒	Estimates of effect sizes (e.g. Cohen's d, Pearson's r), indicating how they were calculated

Our web collection on statistics for biologists contains articles on many of the points above.

Software and code

Policy information about availability of computer code

Data collection	Excel
Data analysis	JASP

For manuscripts utilizing custom algorithms or software that are central to the research but not yet described in published literature, software must be made available to editors and reviewers. We strongly encourage code deposition in a community repository (e.g. GitHub). See the Nature Research guidelines for submitting code & software for further information.

Data

Policy information about availability of data

All manuscripts must include a data availability statement. This statement should provide the following information, where applicable:

- Accession codes, unique identifiers, or web links for publicly available datasets
- A list of figures that have associated raw data
- A description of any restrictions on data availability

The data that support the findings of this study are available from the corresponding author [JB], upon reasonable request.

Field-specific reporting

Please select the one below that is the best fit for your research. If you are not sure, read the appropriate sections before making your selection.

☒ Life sciences ☐ Behavioural & social sciences ☐ Ecological, evolutionary & environmental sciences

For a reference copy of the document with all sections, see nature.com/documents/nr-reporting-summary-flat.pdf

Life sciences study design

All studies must disclose on these points even when the disclosure is negative.

Sample size	Pilot study
Data exclusions	A total of 35 patients were screened for eligibility. Nine patients declined to participate, three did not meet the inclusion criteria and one patient was referred to another treatment (Please see the CONSORT flow chart diagram shown in Figure 1). The final analyzed sample consisted in 22 outpatients
Replication	NA
Randomization	twenty-two patients with schizophrenia were randomized (excel list by the sponsor of the study, block of four) , no stratification
Blinding	patients and investigators (raters) were blinded. Analysis of VAS

Reporting for specific materials, systems and methods

We require information from authors about some types of materials, experimental systems and methods used in many studies. Here, indicate whether each material, system or method listed is relevant to your study. If you are not sure if a list item applies to your research, read the appropriate section before selecting a response.

Materials & experimental systems

n/a	Involved in the study	
☒	☐	Antibodies
☒	☐	Eukaryotic cell lines
☒	☐	Palaeontology and archaeology
☒	☐	Animals and other organisms
☐	☒	Human research participants
☐	☒	Clinical data
☒	☐	Dual use research of concern

Methods

n/a	Involved in the study	
☒	☐	ChIP-seq
☒	☐	Flow cytometry
☐	☒	MRI-based neuroimaging

Human research participants

Policy information about studies involving human research participants

Population characteristics	age, gender, diagnose, severity of symptoms
Recruitment	Psychiatric hospital
Ethics oversight	CPP sud EST

Note that full information on the approval of the study protocol must also be provided in the manuscript.

Clinical data

Policy information about clinical studies

All manuscripts should comply with the ICMJE guidelines for publication of clinical research and a completed CONSORT checklist must be included with all submissions.

Clinical trial registration	NCT00875498
Study protocol	NCT00875498
Data collection	NCT00875498
Outcomes	NCT00875498

Magnetic resonance imaging

Experimental design

Design type	resting-state
Design specifications	2 sessions per subject
Behavioral performance measures	NA

Acquisition

Imaging type(s)	functional
Field strength	1.5
Sequence & imaging parameters	T2*-weighted echo-planar imaging (EPI) sequence with the following parameters: 5 min, 120 volumes, 29 transverse slices, TR = 2500 ms, TE = 50 ms, field of view = 220 mm^2, and voxel size = 3.438 x 3.438 x 4 mm.
Area of acquisition	whole brain

Diffusion MRI ☐ Used ☒ Not used

Preprocessing

Preprocessing software	CONN toolbox (v18.b, https://www.nitrc.org/projects/conn)
Normalization	functional images were segmented and spatially normalized into the standard MNI space (Montreal Neurological Institute, Canada)
Normalization template	MNI
Noise and artifact removal	Denoising included the linear regression of several confounding parameters, including the six motion parameters derived from spatial-motion correction and their first-order derivatives, as well as the BOLD signal from the CSF and white matter masks (5 components each) using the anatomical CompCor approach.
Volume censoring	Motion outliers detected by the artifact detection toolbox were censored.

Statistical modeling & inference

Model type and settings	At the first-level, Fisher's z-transformed Pearson's correlation coefficients were computed between the mean time-series averaged across seed voxels that were within the estimated gray matter mask for each subject and the time course of all of the other voxels.
Effect(s) tested	At the second-level, between-group analyses were performed to compare the left DLPFC functional connectivity changes (post-iTBS minus pre-iTBS) between active and sham groups using two-sample T tests.

Specify type of analysis: ☒ Whole brain ☐ ROI-based ☐ Both

Statistic type for inference (See Eklund et al. 2016)	voxel-wise
Correction	The results were thresholded at an uncorrected voxel-level threshold of $p < 0.001$ with a familywise error (FWE)-corrected p <0.05 at the cluster level to correct for multiple comparisons.

Models & analysis

n/a	Involved in the study
☐	☒ Functional and/or effective connectivity
☐	☐ Graph analysis
☐	☐ Multivariate modeling or predictive analysis

Functional and/or effective connectivity	Pearson's correlations
Graph analysis	N/A
Multivariate modeling and predictive analysis	N/A
